# Stable Expression of Recombinant Factor VIII in CHO Cells Using Methotrexate-Driven Transgene Amplification

**Published:** 2012

**Authors:** N. A. Orlova, S. V. Kovnir, I. I. Vorobiev, A.S. Yuriev, A.G. Gabibov, A.I. Vorobiev

**Affiliations:** Shemyakin and Ovchinnikov Institute of Bioorganic Chemistry, Russian Academy of Sciences; Hematology Research Centre Ministry of Healthcare and Social Development of the Russian Federation

**Keywords:** coagulation factor VIII, B-domain deleted factor VIII, hemophilia A, heterologous protein expression systems

## Abstract

Prophylaxis and treatment of inherited clotting disorder hemophilia A requires regular administration of factor VIII. Recombinant factor VIII, which is produced in CHO or BHK cells, is equivalent to the plasma-derived one and is prevalent in current clinical practice in developed countries. Development of a biosimilar recombinant FVIII requires the creation of a highly productive clonal cell line and generation of monoclonal antibodies suitable for affinity purification of the product. Methotrexate-driven transgene amplification of genetic cassettes that code full-length and truncated variants of FVIII under the control of the CMV promoter was studied. It was shown that the expression level of the truncated variant of FVIII is 6.5 times higher than that of the full-length molecule. The transgene amplification procedure was sufficient for a twofold increase of the expression level in the transfected cells pool and subsequent selection of the clonal line, stably producing truncated FVIII at the level of 0.52 IU/ml during cultivation in a chemically defined protein-free culture medium. Four generated mouse monoclonal antibodies toward the heavy chain of FVIII were found suitable for binding the truncated variant of FVIII directly from the conditioned medium and elution of the FVIII with a more than 85% yield and normal pro-coagulant activity. The producer cell line and monoclonal antibodies obtained are sufficient for the development of upstream and downstream processes of biosimilar FVIII production. Generation of more productive cell lines by the use of stronger, nonviral promoters and shorter cDNA of FVIII will be the subject of further studies.

## Introduction

Blood clotting factor VIII (FVIII) is a nonenzymatic cofactor for factor IXa forming a complex that binds factor X and activates it, realizing a major amplification loop of the blood coagulation cascade. Defects in the gene of FVIII result in hemophilia A, a recessive X-linked coagulation disorder with a prevalence of 1 case per 5,000 males.

Protein substitution therapy, performed on a regular basis, is the only effective treatment for hemophilia A. The traditional source of FVIII is donated blood plasma, which is in short supply and poses a significant risk of virus [1, 2] and prions [3] transmission, even after rigorous plasma batches screening and multiple viral inactivation procedures. Recombinant human FVIII (rhFVIII) for hemophilia A treatment may be obtained from cultured mammalian cells purified to clinical grade by affinity chromatography and three or four rounds of conventional chromatography and virus-inactivated by solvent-detergent treatment and nanofiltration or heating. Marketed variants of rhFVIII are expressed in Chinese hamster ovary (CHO) or baby hamster kidney (BHK) cells and are fully equivalent to the plasma-derived FVIII in replacement therapy.

**Table 1:** Primers used for FVIII-SQ BDD mutant construction. Restriction sites are underlined

O1KpnIfor	5’GCTGGTACCTCACAGAGAATATACA3’
O1HindIIIrev	5’GGAGAAGCTTCTTGGTTCAATG3’
O2HindIIIfor	5’CCAAGCTTCTCCCAAAACCCACCA GTCTTGAAAC3’
O2BlpIrev	5’CTGCCCATGCTGAGCAGATAC3’
Odelf	5’GCCACAACTCAGACTTTCG3’
8sq4f	5’TGTATTTGATGAGAACCGAAGC3’
8sq5r	5’GCCACTCTGAGCCCTGTT3’
CMVfor	5’CGCAAATGGGCGGTAGGCGTG3’
8sq15r	5’GAGTTCTTTGTTTCTGAGTGCC3’

**Table 2:** Primers used for sequence analysis of FVIII ORF

8sq1f	TGATCAGACCAGTCAAAGGGA
8sq2f	GATTGGATGCCACAGGA
8sq3f	GCCCTCAGCGGATTGGT
8sq4f	TGTATTTGATGAGAACCGAAGC
8sq5f	TGCCATTGAACCAAGAAGC
8sq6f	GAGAAACTGGGGACAACTGC
8sq7f	AGAAAGACTCACATTGATGGCC
8sq8f	ACAAAGTGGTAGTAGGAAAGGGTG
8sq9f	TGAAACAATTCAGACTCCCACT
8sq10f	GACAAGTGCCACAAATTCAG
8sq11f	TTTGTCCCTGAACGCTTGT
8sq12f	CAGCCCTTATACCGTGGAG
8sq13f	CAGATGGAAGATCCCACTTT
8sq14f	GGATCAATCAATGCCTGGAG
8sq15f	AGGAGTAATGCCTGGAGACC
8sq1re	GCAAGCCAGGGAGGGAC
8sq2re	TGGCAAACATATTGGTAAAGTA
8sq3re	AGGGGAGTCTGACACTTATTGC
8sq4re	GAGCAAATTCCTGTACTGTCACTT
8sq5re	GCCACTCTGAGCCCTGTT
8sq6re	CTTGGGATTTCCACTCTTCTTT
8sq7re	CTGCTGGAAGATGAGAAGAGTT
8sq8re	TGCTGGCTTGTATTAGGAGA
8sq9re	GCCTTGCCCAGAGTTCAG
8sq10re	AGTCAACAAAGCAGGTCCAT
8sq11re	ACTGTCTATTGCTCCAGGTGA
8sq12re	CTGAGAATGGGAATAGGGTGA
8sq13re	GGGTCAGGCACCGAGGA
8sq14re	GGATGCTTCTTGGCAACTGA
8sq15re	GAGTTCTTTGTTTCTGAGTGCC
IRESArev	AGGTTTCCGGGCCCTCACATTG

The major drawback of rhFVIII production techniques is the low expression level, which is caused by the unusual size and structural complexity of the target protein. Natural human FVIII is a 170- to 280-kDa glycoprotein, mainly present in circulation in the form of a noncovalent complex with the chaperone - von Willebrand factor (vWF) in a concentration of approximately 400 ng/ml. FVIII is expressed in the liver as a single-chain polypeptide containing the A1-A2-B-A3-C1-C2 domains. The mature, secreted protein is cleaved in the region between the B and A3 domains and forms a heterodimer of 90-200 kDa, heavy chain (A1-A2-B domains), and 80 kDa, light chain (A3-C1-C2 domains) [4]. A significant part of the FVIII molecule, the B-domain, may be deleted without compromising the clotting activity and plasma half-life of the truncated FVIII [5]. Replacement of the B-domain by the short linker peptide called SQ results in a significant increase in the rhFVIII expression level in CHO cells and the complete processing of the precursor protein to its mature form [6]. The B-domain-deleted FVIII (BDD-FVIII), which is marketed under the trade name ReFacto, has shown comparable efficiency with full-length FVIII variants and is as safe [7]. 

The aim of the present study was to generate a clonal cell line secreting rhFVIII at a significant level and to develop monoclonal antibodies for affinity purification of rhFVIII. Since the ability of cell lines bearing a single-copy genomic insert to produce enough rhFVIII has not been confirmed in published studies, FVIII or BDD-FVIII coding vectors suitable for insertion cassette amplification were evaluated.

## Experimental Part

**Construction of expression plasmids: **

For pOptivec/F8 construction, pOptivec plasmid derived from a re-ligated linear pOptivec-TOPO vector (Invitrogen, USA) was restricted by *NotI* and ligated by T4-ligase with a *NotI-NotI* fragment of pCMV6-XL4/NM_000132 containing the full factor VIII gene (Origene, USA). The enzymes used were acquired from Fermentas, Lithuania, or Sibenzyme, RF.

For BDD-FVIII generation, the PCR fragments F1 (479 b.p.) and F2 (933 b.p.) that flank the deleted region were obtained using the primers O1KpnIfor, O1Hindrev and O2Hindfor, and O2Blprev, respectively (Supplementary  *Table 1* ). Oligonucleotides were synthesized by Evrogen JSC, RF. PCR was performed by a Tersus polymerase mix (Evrogen JSC, RF) on the PTC-100 Thermal Cycler (MJ Reseach, USA); purified PCR products were cloned to the pAL-TA vector (Evrogen JSC, RF) and fully sequenced using the BigDye Terminator v. 3.1 cycle sequencing kit (Applied Biosystems, USA), a ABI PRISM 3730 genetic analyzer (Applied Biosystems, USA), and the Chromas 1.45 program (Technelysium Pty Ltd, Australia) for data analysis.

The N-terminal FVIII gene fragment F3 was obtained by pCMV6-XL4/NM_000132 restriction with the *NotI* and *KpnI* enzymes. Assembly of the fragments F1-3 was performed in the PAL-TA vector by corresponding restriction enzymes resulting in pALTA/F123. PCR for clone analysis was performed with the Odelf specific primer and the vector-specific M13for and M13rev primers. 

The * BlpI-BlpI* fragment of the pOptivec/F8 plasmid was exchanged for the *BlpI-BlpI* restriction fragment of pALTA/F123, resulting in the pOptivec/F8BDD plasmid. PCR for clone analysis was performed with two specific primer pairs: 8sq4f, 8sq5r and CMVfor, and 8sq15r. The ORFs of full-length FVIII and BDD-FVIII and expression vector functional elements (promoter, IRES, terminator) were sequenced using the specific primers listed in Supplementary Table 2.

Preparation of the assembled plasmids for transfection was done by transformation to a Stbl4 *E.*   *coli* strain (#11635018 Invitrogen, USA), cultivation in a 0.5 L TB broth for 18 h, and purification by the EndoFree Plasmid MaxiKit (Qiagen, USA). For stable cell lines generation, plasmids were linearized by *PvuI* restriction, followed by ethanol precipitation. The precipitates were dissolved in PBS and filter-sterilized using 0.22 µm filters (Millipore, USA).

Cell culture: A DHFR-negative Chinese Hamster Ovary CHO DG-44 cell line (Invitrogen, USA) maintained in a chemically defined suspension medium was used. The cells were cultivated in a suspension culture as 30 mL batches in Erlenmeyer flasks (VWR, USA), with a CD DG-44 medium (Invitrogen, USA) supplemented with 8 mM L-glutamine (Invitrogen, USA) and 0.18% Pluronic F-68 (BASF Inc., USA). The culture flasks were maintained in a humidified incubator, 37°C/8% CO _2_ on a shaker, at a constant rotation rate of 130 rpm. Viability by trypan blue exclusion assay was assessed and cell count performed at each cell passage. Cells were passaged every 2-3 d and maximum cell concentration was set at 1.2x10 ^6^  viable cells in 1 ml: split ratio 1:4. 

Transfection and selection of stably transfected cells: transfection was performed by the animal origin free reagent Lipofectamine 2000 (Invitrogen, USA) using 18 µg of linearized plasmid DNA per 1,5x10 ^7^  cells in 30 mL of the culture medium. Cells were cultivated 48 h post-transfection without medium change, then they were transferred to the selection nucleoside-free medium CD OptiCHO (Invitrogen) supplemented with 8 mM L-glutamine (Invitrogen, USA) and cultivated until cell viability reached 90% (10-20 d). During cultivation in the selection medium, the cells were passaged every 3 d or at a concentration of 3x10 ^5^ cells/mL. The levels of FVIII secretion were determined 48 h after transfection and at the end of cultivation in the selection medium. Three independent transfections were performed in the same conditions for each plasmid. The highest producing pool was selected for the methotrexate (MTX) induced amplification of *dhfr*  and the FVIII genes.

Clonal cell lines generation: the selected pool of stably transfected cells was subjected to growth in the presence of increasing concentrations of methotrexate in a CD OptiCHO medium supplemented with 8 mM L-glutamine. At every subsequent step of the MTX-driven target gene amplification, the concentration of MTX was increased twofold. On each step, the cells were cultivated for at least 10 days, then 4 to 15 more days until cell viability reached 90%. The levels of secreted FVIII were measured by ELISA at the end of each step. The highest producing amplified pool was used for obtaining clonal cell lines by limiting dilution at 0.5 cells/well. Cloning was performed in the adherent culture, utilizing a medium CD CHO-A (Invitrogen, USA) (200 µl/well) containing 8 mM GlutaMAX (Invitrogen, USA) at 37°C/ 5% CO _2_ for 21 days. MTX was excluded from the cloning medium and was not used in further cultivation. 

The growth of single colonies in wells was monitored and documented on days 10 and 14. The colonies were transferred to 48-well plates, and the conditioned medium from the wells with actively grown colonies was assayed by ELISA. The best secreting cell clones were further propagated in the adherent conditions and re-adapted to the suspension culture in 3 consecutive passages in 24-, 12-, and 6-well plates, utilizing a CD OptiCHO medium with 8 mM L-glutamine. The conditioned medium from 6-well plates was screened by ELISA; one clonal line was selected and expanded further by subsequent passages in 3, 15, 100, and 200 mL of CD OptiCHO.

Small-scale production culture was done in shaking flasks at a 200-mL scale. Cells were seeded at 2,5x10 ^5^  cells/mL, cultured without medium change to a density of 3x10 ^6^  cells/mL (4-5 days), and then cultured for 3 more days with a daily addition of 4 mM glutamine and 3 mM glucose. Cell mass and debris were removed by centrifugation at 500 g for 5 min and subsequent filtration of supernatant by 0, 22 µm PES capsule filter (Millipore, USA). The clarified medium was stored frozen and thawed immediately before use. 

ELISA: ELISA was performed as described in [8]. Antibody capture ELISA was used for the testing of anti FVIII mAbs, and a concentrate of plasma-derived FVIII (a generous gift from Dr. A.L. Berkovsky) in PBS at 200 ng/well was used for plate coating. Sandwich ELISA was utilized for secreted FVIII in the culture medium, polyclonal anti-FVIII antibodies (LifeSpan BioSciences, USA) at 50 ng/well were used for plate coating, and in-house developed anti-FVIII murine mAb A2 was used for detection. Frozen pooled normal human plasma serially diluted in 1% BSA-PBS was used as a quantity calibrator. All the samples tested were applied to plates undiluted or diluted immediately before testing by 1% BSA-PBS. 

Western blotting: Whole-cell lysates were prepared with a modified RIPA buffer (50 mM Tris-HCl, pH 7.4; 1% NP-40; 0.25% Na-deoxycholate, 150 mM NaCl, 1 mM EDTA) containing a protease inhibitor cocktail (Sigma, USA). Samples of the conditioned medium were clarified by centrifugation and concentrated 30-fold by trichloroacetic acid precipitation. The samples were normalized by total protein concentration, applied at 10 µg of total protein per lane, and resolved on 7.5% SDS-PAGE gels. Protein transfer, blocking, hybridization, and color development were done according to [8] using a Hybond C Extra membrane (GE Healthcare, USA) and a 3,3’,5,5’-tetramethylbenzidine substrate (Sigma, USA). 

Generation of mAbs: Immunization, fusion, and cloning of hybridomas were performed according to [8]. Female Balb/c mice (Harlan Labs, UK) were immunized subcutaneously with 100 ng of recombinant full-length FVIII (Kogenate FS) in 0.25 mL of 0.85% NaCl and 0.25 mL of complete Freund’s adjuvant. (Pierce Biotechnology, USA). Two and four weeks after the initial injection, the animals were boosted with 100 ng of the same antigen in IFA. One week after the last injection, the mice were tail-bled and the serum antibody level was monitored by ELISA. One mouse with the highest titer of IgG was sacrificed for cell fusion. Splenocytes from the chosen mouse were fused with SP2/0 myeloma cells using polyethylene glycol. The fused cells were propagated in a selective medium, plated in 96-well plates, and then screened for anti-fVIII IgG titer. The cells from positive wells were expanded to 24-well plates and screened for titer and sensitivity to elution by 50% ethylene glycol in PBS. The wells with the highest titers and highest sensitivity to ethylene glycol elution were used for hybridoma cloning by limiting dilution (0.5 cells/w). Expanded hybridoma clones were re-screened by the same procedure and cloned again. The expanded clones were used for generation of the conditioned medium (10-100 mL) and ascites production in pristane-primed Balb/c mice. The ascitic fluid collected was stored frozen for further use. 

Purification of monoclonal antibodies from the ascitic fluid and conditioned medium was performed by the same protocol – precipitation by ammonium sulphate, Protein G affinity chromatography using a HiTrap Protein G HP (GE Healthcare, USA) 1 ml column, concentration of eluted IgG by ultrafiltration, and polishing/desalting by size exclusion chromatography utilizing a Superdex 75 10/300 column (GE Healthcare, USA) and PBS as the mobile phase.

NHS-activated Sepharose 4 Fast Flow (GE HealthCare, USA) was used for mAb’s coupling according to the resin manufacturer`s instruction. Antigen capture was performed in batch format, and 1 ml aliquots of the conditioned medium was mixed in microcentrifuge test tubes with 0.1 ml aliquots of affinity sorbents for 1 h at room temperature. Sorbents were settled by brief centrifugation, and the supernatants of the depleted medium were collected for further analysis. Sorbents were washed by 3 1 ml portions of PBS; bound proteins were eluted by the addition of 0.15 ml of 50% ethylene glycol in the PBS solution and 5 min incubation. 

Coagulation assay: The clotting assay for the fVIII level was performed on a ThromboScreen 400c (Pacific Hemostasis A Fisher Scientific Company) optical coagulometer using the reagents kit “Factor VIII-test” (NPO Renam, RF) according to the kit manufacturer’s protocol with some modifications. Culture media samples were diluted ten times by imidazole solution prior to the analysis, and eluates from the affinity columns were diluted 10-50 times by a imidazole solution supplemented with 1% BSA. For testing the conditioned media samples, calibration plasma samples were supplemented by 10% of the conditioned medium from non-transfected CHO DG-44 cells. In case of affinity column eluates, the calibration samples were supplemented by 2-10% of the elution solution.

## Results and discussion

**Fig. 1:**
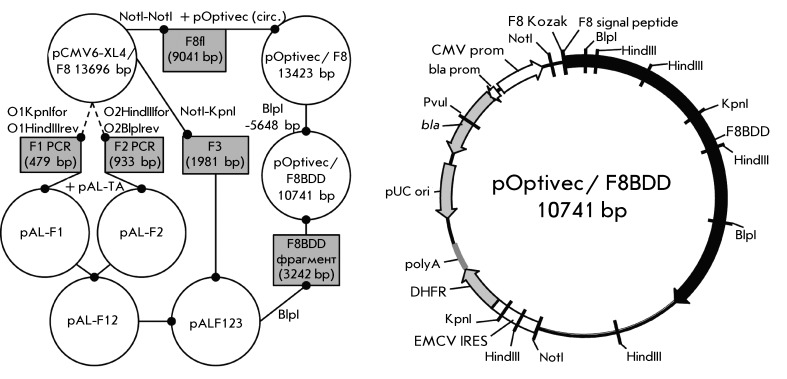
General molecular cloning scheme and map of the pOptivec/F8BDD expression plasmid. Linear fragments shown as rectangles, plasmids – as circles. PCR steps are shown as dotted lines, restriction-ligation steps – as full lines. CMV-prom - cytomegalovirus promoter, F8 Kozak - natural Kozak sequence of the FVIII gene, F8 signal peptide – natural FVIII signal peptide sequence, F8BDD – ORF of the SQ B-domain deleted factor VIII, EMCV IRES - encephalomyocarditis virus internal ribosome entry site, dhfr - ORF of the dihydrofolate reductase, polyA – bovine growth hormone polyadenylation signal, pUC ori – bacterial replication origin, bla – ampicillin resistance gene, bla prom - ampicillin resistance gene promoter. Directions of genes transcription are shown by arrows. Restriction sites used for cloning procedures are in italics.

An expression construct of the full-length factor VIII gene pOptivec/F8 was created on the base of the pOptivec-TOPO vector. The SQ B-domain deletion mutant cloning strategy included minimization of the PCR fragments length to bypass PCR-mediated mutations. Two short PCR fragments, F1 and F2, flanking the deleted region were separately cloned and then assembled with F3 - a restriction fragment of pCMV6-XL4/NM_000132 corresponding to the N-terminal part of the FVIII protein. To obtain the expression plasmid, the pOptivec/F8BDD *BlpI-BlpI* fragment of the pOptivec/F8 expression plasmid was exchanged for the *BlpI-BlpI* fragment of the obtained F123 assembly ( *Fig. 1* ). 

The resulting expression plasmid contained a strong CMV-promoter, a natural FVIII Kozak sequence, F8-BDD ORF, followed by a encephalomyocarditis virus (EMCV) internal ribosome entry site (IRES) that allows 5’ cap-independent translation initiation: the dhfr gene which allows clonal selection of transfectants and further MTX-driven amplification of the fVIII-IRES-dhfr cassette in *dhfr-* cells. For stable cell lines generation plasmids were linearized for destruction of the irrelevant ampicillin resistance gene. 

**Generation of the producer cell line**

Transfection of DHFR-negative CHO DG-44 cells by linearized expression cassettes was performed in a serum-free medium utilizing an animal-origin free transfection reagent. Transfection efficiency was estimated by transfection of the control plasmid coding fluorescent eGFP protein; 10 to 20% of the cells expressed eGFP at 48 h post-transfection, and cell viability was above 85%. Three rounds of transfection were performed for each FVIII-coding plasmid, and stable pools were obtained by cultivation of the transfected cells in a selective medium for 15-20 days. The levels of secreted FVIII were determined by ELISA; in the case of full-length FVIII, less than 10 IU/l of secreted FVIII was found in all cell pools, and in the case of BDD-FVIII, a stable transfectant pool with a 71±10 IU/ml secretion level was found and used for transgene amplification. 

**Fig. 2:**
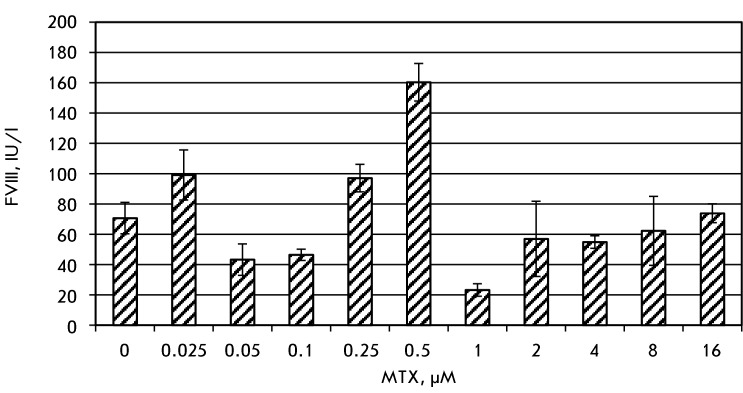
Secretion level of BDD-FVIII for confluent cell pools by ELISA. Samples in duplicates, error bars represent a 95% confidence interval.

The pool of stably transfected cells was treated by MTX, starting at the 25 nM level. After stabilization of the culture, determined as improvement in cell viability to more than 85%, the concentration of MTX was increased twofold and amplification continued ( *Fig. 2* ). It was found that a steady increase in the secreted BDD-FVIII level does not take place; the maximum BDD-FVIII level in the conditioned medium was attained after 5 subsequent amplification steps (0,5 µM of MTX). Further increase of MTX concentration resulted in an immediate tenfold drop in the product secretion rate and subsequent increase to the values of the initial culture (74±6  IU/l at 16 µМ MTX). It was suggested that non-producing cells containing altered bicistronic mRNA [9] or amplified non-relevant genes, for example the gene of multiple-drug resistance protein 1 [10], take over the producing population. 

The best producing cell pool, obtained at the level of 0.5 µM MTX, was used for the generation of clonal cell lines by limiting dilution. Twenty-two clones derived from the wells with single cell colonies and secreting the target protein according to ELISA were grown to more than 10 ^6^  cells in the adherent culture conditions and analyzed for the concentration of secreted FVIII ( *Table 1* ). Four clones with the highest production rate were further cultivated in the suspension culture and tested again for the concentration of FVIII after three and ten consequent passages (10 and 30 days of continuous culturing). Two clones, #18 and #22, showed no significant drop in FVIII production (data not shown), and the more productive clone #18 was chosen for subsequent use. 

**Table 3:** Productivity of the 10 highest secreting clonal cell lines. Product concentration was measured for adherent cultures at the attainment of confluence

Clone number	18	22	17	9	1	2	15	3	4	16
Secreted FVIII , IU/l	502	475	434	416	410	399	395	379	378	375

**Fig. 3:**
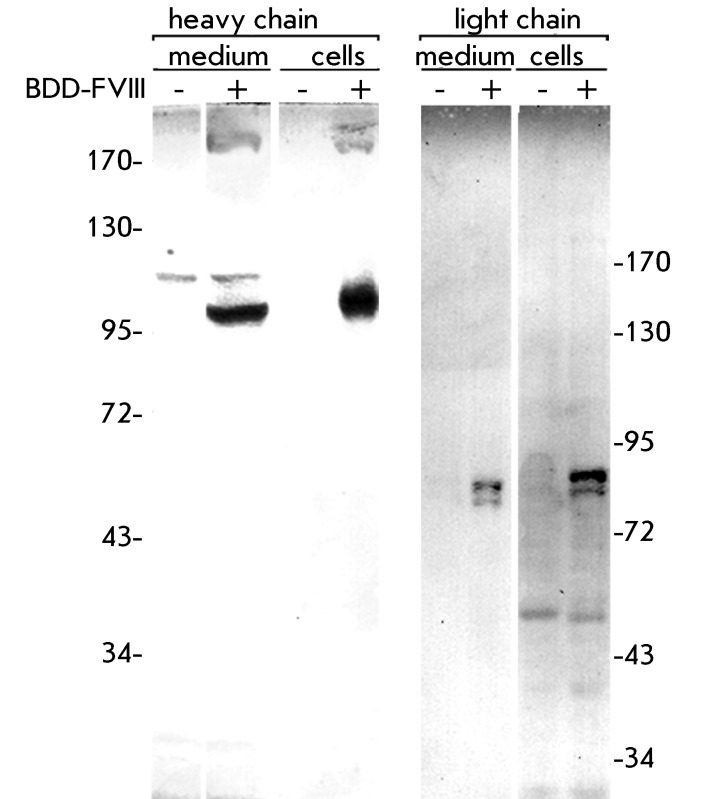
Western blotting of secreted and intracellular BDD-FVIII. “Heavy chain” and “light chain” – probing by antibodies toward heavy and light chain, “BDD-FVIII -” and “BDD-FVIII +” – samples from nontransfected CHO DG-44 cells and cell line DG-BDDFVIII-18, respectively. SDS-PAGE in reducing conditions, molecular weights are shown in kDa.

The conditioned medium from the chosen clonal cell line, designated DG-BDDFVIII-18, was used for the characterization of the secreted FVIII. According to Western blotting data ( *Fig. 3* ), the bulk of the secreted FVIII, as well as the intracellular precursor, was processed into the form of a two-chain protein. Low levels of the single-chain form were detectable only by the antibody toward the heavy chain, and no degradation products were detected by both antibodies, which is an indication of the proper short-term stability of FVIII in the culture medium used. 

The pro-coagulant activity of FVIII in the conditioned medium was assessed based on its ability to decrease the clot time of the substrate plasma obtained from hemophilia A patients in the aPTT test. The activity of FVIII in the conditioned medium tested was 0.47 IU/ml, and the FVIII antigen level in the same sample was 0.52 IU/ml according to ELISA data; hereby, the recombinant FVIII secreted by the clonal cell line DG-BDDFVIII-18 has full specific activity.

The known industrial purification process for the BDD-FVIII protein consists of 4 stages of conventional chromatography and one round of affinity purification [11]. Therefore, the key element in the process is the monoclonal antibody, which is capable of capturing BDD-FVIII from the culture medium or from the intermediate concentrate and of subsequently releasing the product under mild elution conditions. The typical solution suitable for the elution of FVIII from immunoaffinity columns is PBS with 50% ethylene glycol [12].

A monoclonal antibody suitable for the affinity purification of BDD-FVIII was obtained by screening anti-FVIII hybridoma clones by ELISA, in which the wells were washed three times with a 50% ethylene glycol solution after the incubation of hybridoma culture supernatants and the control wells were washed with a PBS-Tween solution. The target clone is expected to show a significant signal decrease upon ethylene glycol wash. Out of the 34 individual clones derived from one cell fusion, four clones with a high mAb titer and the highest sensitivity to ethylene glycol wash were selected ( *Table 3* ). 

All of the four mAbs selected recognized the heavy chain of BDD-FVIII on western blotting; i.e., their epitopes do not belong to the B-domain of FVIII. Purified mAbs were obtained from the ascitic fluid and immobilized on NHS-activated Sepharose at 1 mg of mAb per 1 ml of the settled resin ratio. Immunosorbents were used to capture BDD-FVIII directly from the conditioned culture medium with 1 h batch adsorption. Adsorption of BDD-FVIII in these conditions was incomplete (ca. 20-30%), but nearly the entire bound product was retained notwithstanding PBS wash and eluted with the 50% ethylene glycol solution. The levels of FVIII in the nonbound fractions and eluates were measured by plasma clot assays. The presence of biologically active FVIII in the eluates indicates that the elution conditions used did not significantly degrade the product. Thus, several mAbs suitable for large-scale affinity purification of recombinant FVIII were obtained.

## Conclusions

**Table 4:** Properties of ethylene glycol-sensitive mAbs

Clone name	mAb titer in the ascitic fluid	Decrease in ELISA signal at ethylene glycol wash, %	Resin binding capacity, IU/ml	FVIII elution degree, %
A2	1:123 000	40%	2.6	89%
E3	1:68 500	39%	2.8	89%
A4	1:27 500	15%	1.6	>90%
B6	1:123 000	35%	3.4	86%

The purpose of this study was to develop a recombinant FVIII producing stable cell lines and monoclonal antibodies for affinity purification of the secreted protein.

Expression constructs bearing completely sequence-verified cDNA’s of full-length human FVIII and a B-domain deletion variant of human FVIII were obtained. Stably transfected cell pools were obtained; the BDD-FVIII variant showed a vastly increased level of secretion and was used for methotrexate-driven transgene amplification and subsequent cell cloning. A clonal cell line DG-BDDFVIII-18, capable of stable secretion of BDD-FVIII at the 500 IU/l level, was established. The target protein in the conditioned medium was found to be biologically active and almost entirely processed into its two-chain mature form. The cell line was obtained without the use of animal-origin substances and stably grows in a chemically defined medium as a suspension culture. Monoclonal antibodies toward the heavy chain of BDD-FVIII, suitable for affinity purification of the target protein in native form, were obtained. 

Thus, both of the major components of the industrial FVIII production process were created – the animal-origin free clonal cell line and monoclonal antibodies for the affinity purification step. Generation of more productive clonal cell lines and overall FVIII production process development will be studied subsequently. 

## Abbreviations

FVIII: blood clotting factor VIII
rhFVIII: recombinant human FVIII, BDD-FVIII – FVIII with deleted B-domain, MTX ‑ methotrexate, EG ‑ ethylene glycol, EMCV ‑ Encephalomyocarditis virus, IRES ‑ internal ribosome entry site, DHFR ‑ dihydrofolate reductase (EC 1.5.1.3)
IU: international unit (1 IU of FVIII corresponds to it's content in 1 ml of pooled donor plasms), ORF – open reading frame, PBS – phosphate buffered solution

## References

[1] Blumel J., Schmidt I., Effenberger W., Seitz H., Willkommen H., Brackmann H.H., Lower J., Eis-Hubinger A.M. (2002). Transfusion..

[2] Yokozaki S., Fukuda Y., Nakano I., Katano Y., Toyoda H., Takamatsu J. (1999). Blood..

[3] Evatt B.L. (1998). Haemophilia..

[4] Thompson A.R. (2003). Semin. Thromb. Hemost..

[5] Pittman D.D., Alderman E.M., Tomkinson K.N., Wang J.H., Giles A.R., Kaufman R.J. (1993). Blood..

[6] Lind P., Larsson K., Spira J., Sydow-Backman M., Almstedt A., Gray E., Sandberg H. (1995). Eur. J. Biochem..

[7] Kessler C.M., Gill J.C., White G.C., Shapiro A., Arkin S., Roth D.A., Meng X., Lusher J.M. (2005). Haemophilia..

[8] Chun B.H., Park S.Y., Chung N., Bang W.G. (2003). Biotechnol. Lett..

[9] Harlow E., Lanes D. (1988). Antibodies: A laboratory manual. Cold Spring Harbor. N.Y.; Cold Spring
Harbor Lab. Press,.

[10] Fann C.H., Guirgis F., Chen G., Lao M.S., Piret J.M. (2000). Biotechnol. Bioeng..

[11] Assaraf Y.G., Molina A., Schimke R.T. (1989). J. Biol. Chem..

[12] Kelley B.D., Booth J., Tannatt M., Wub Q.L., Ladner R., Yuc J., Potter D., Ley A. (2004). J. Chromatogr. A..

[13] Kelley B., Jankowski M., Booth J. (2010). Haemophilia..

[14] Griffith M. (1991). Ann. Hematol..

